# AI-predicted mpMRI image features for the prediction of clinically significant prostate cancer

**DOI:** 10.1007/s11255-023-03722-x

**Published:** 2023-08-09

**Authors:** Song Li, Ke-Xin Wang, Jia-Lei Li, Yi He, Xiao-Ying Wang, Wen-Rui Tang, Wen-Hua Xie, Wei Zhu, Peng-Sheng Wu, Xiang-Peng Wang

**Affiliations:** 1 Zhejiang Chinese Medical University, China, The Affiliated Hospital of Jiaxing University, Jiaxing, China; 2 School of Basic Medical Sciences, Capital Medical University, Beijing, China; 3 The Affiliated Hospital of Jiaxing University, Jiaxing, China; 4 Department of Radiology, Peking University First Hospital, Beijing, China; 5 Beijing Smart Tree Medical Technology Co. Ltd., Beijing, China

**Keywords:** Gleason score, Prostate cancer, Multiparametric magnetic resonance imaging, Deep learning, International Society of Urological Pathology grade group

## Abstract

**Purpose:**

To evaluate the feasibility of using mpMRI image features predicted by AI algorithms in the prediction of clinically significant prostate cancer (csPCa).

**Materials and methods:**

This study analyzed patients who underwent prostate mpMRI and radical prostatectomy (RP) at the Affiliated Hospital of Jiaxing University between November 2017 and December 2022. The clinical data collected included age, serum prostate-specific antigen (PSA), and biopsy pathology. The reference standard was the prostatectomy pathology, and a Gleason Score (GS) of 3 + 3 = 6 was considered non-clinically significant prostate cancer (non-csPCa), while a GS ≥ 3 + 4 was considered csPCa. A pre-trained AI algorithm was used to extract the lesion on mpMRI, and the image features of the lesion and the prostate gland were analyzed. Two logistic regression models were developed to predict csPCa: an MR model and a combined model. The MR model used age, PSA, PSA density (PSAD), and the AI-predicted MR image features as predictor variables. The combined model used biopsy pathology and the aforementioned variables as predictor variables. The model’s effectiveness was evaluated by comparing it to biopsy pathology using the area under the curve (AUC) of receiver operation characteristic (ROC) analysis.

**Results:**

A total of 315 eligible patients were enrolled with an average age of 70.8 ± 5.9. Based on RP pathology, 18 had non-csPCa, and 297 had csPCa. PSA, PSAD, biopsy pathology, and ADC value of the prostate outside the lesion (ADC_prostate_) varied significantly across different ISUP grade groups of RP pathology (*P* < 0.001). Other clinical variables and image features did not vary significantly across different ISUP grade groups (*P* > 0.05). The MR model included PSAD, the ratio of ADC value between the lesion and the prostate outside the lesion (ADC_lesion/prostate_), the signal intensity ratio of DWI between the lesion and the prostate outside the lesion (DWI_lesion/prostate_), and the ratio of DWI_lesion/prostate_ to ADC_lesion/prostate_. The combined model included biopsy pathology, ADC_lesion/prostate_, mean signal intensity of the lesion on DWI (DWI_lesion_), DWI signal intensity of the prostate outside the lesion (DWI_prostate_), and signal intensity ratio of DWI between the lesion and the prostate outside the lesion (DWI_lesion/prostate_). The AUC of the MR model (0.830, 95% CI 0.743, 0.916) was not significantly different from that of biopsy pathology (0.820, 95% CI 0.728, 0.912, *P* = 0.884). The AUC of the combined model (0.915, 95% CI 0.849, 0.980) was higher than that of the biopsy pathology (*P* = 0.042) and MR model (*P* = 0.031).

**Conclusion:**

The aggressiveness of prostate cancer can be effectively predicted using AI-extracted image features from mpMRI images, similar to biopsy pathology. The prediction accuracy was improved by combining the AI-extracted mpMRI image features with biopsy pathology, surpassing the performance of biopsy pathology alone.

## Background

Prostate cancer (PCa) is the most common malignant tumor among men in Europe and America, and the second leading cause of cancer-related deaths. Early detection of PCa is crucial for proper diagnosis and treatment. The 2017 European Association of Urology Prostate Cancer Guidelines [1] recommend radical therapy as a definitive treatment for intermediate- and high-risk patients with clinically significant prostate cancer (csPCa), defined as having an International Society of Urological Pathology Gleason grade group (ISUP GGG) of 2 or higher. Patients with very low- or low-risk disease are classified as having clinically insignificant prostate cancer (non-csPCa), defined as having a GGG of 1 or some moderately favorable risk (GGG 2), and are recommended to undergo active surveillance without any definitive treatment. Therefore, accurate identification of csPCa versus non-csPCa is critical when determining treatment options.

Currently, prostate cancer is typically diagnosed by conducting a biopsy on men who have elevated levels of serum prostate-specific antigen (PSA) and/or exhibit abnormal digital rectal examination (DRE) results. The biopsy result is then used to determine the aggressiveness of csPCa by measuring the ISUP grading group. However, due to the multifocal and heterogeneous nature of prostate cancer and random undersampling of the entire prostate, conventional 12-core systematic biopsy can produce a false negative rate of up to 30%. To improve the detection of csPCa, a technique called image fusion-guided biopsy is used, which combines mpMRI and ultrasound images to guide needle placement for prostate biopsy. This technique has been shown to improve the detection of csPCa compared to conventional systematic biopsy. However, it is still possible to underestimate csPCa, even when using mpMRI guidance during biopsy.

Over the past few decades, the use of mpMRI in the detection and staging of prostate cancer has become increasingly common. It can help to determine which men with elevated PSA levels should undergo biopsy, which can reduce unnecessary biopsies and increase the sensitivity of detecting csPCa [2]. Additionally, mpMRI has shown potential for predicting the Gleason score with moderate to high accuracy, particularly for csPCa with a Gleason score of 3 + 4 or higher [3]. Therefore, it is reasonable to assume that incorporating mpMRI can improve the accuracy of detecting csPCa in patients initially diagnosed with non-csPCa based on biopsy results. However, interpreting mpMRI for the detection and characterization of PCa requires specialized training and expertise in radiology and prostate cancer.

Using artificial intelligence (AI) methods, such as deep learning (DL), can improve the detection and classification of PCa on mpMRI images [4]. Previous studies have shown that AI can improve the accuracy and efficiency of PCa detection on mpMRI images by automatically detecting and segmenting suspicious areas for further evaluation by a radiologist [5]. Several recent studies have indicated the potential of AI in predicting tumor invasiveness of biopsy pathology [6–8]. Some researchers have also proposed the use of handcrafted or deep radiomics image features for predicting tumor invasiveness [9, 10]. However, there are a limited number of studies that have specifically evaluated tumor invasiveness in the post-biopsy assessment, taking into account both biopsy pathology results and mpMRI findings. Thus, in this study, we explored the feasibility of using mpMRI image features predicted by AI algorithms in the prediction of csPCa in comparison and in combination with biopsy pathology.

## Materials and methods

### Data enrollment

This retrospective study was approved by the institutional review board (IRB number: 2022-LY-361), which waived written patient informed consent. The data were retrospectively gathered from our hospital.

Patients who received prostate mpMRI and subsequent RP between November 2017 and December 2022 were included. The mpMRI images and clinical information, including age, PSA, GS of the biopsy pathology, and GS of the RP, were obtained from the picture archiving and communication system (PACS) and the electronic medical record (EMR) system. The exclusion criteria were (1) prior endocrine therapy, (2) benign prostate hyperplasia on RP pathology, (3) missing PSA data, (4) incomplete biopsy pathology records, (5) incomplete MR images, (6) obvious image artifacts, and (7) prostate cancer volume < 0.5 cm^3^ on MR images.

### Reference standard

**Fig. 1:**
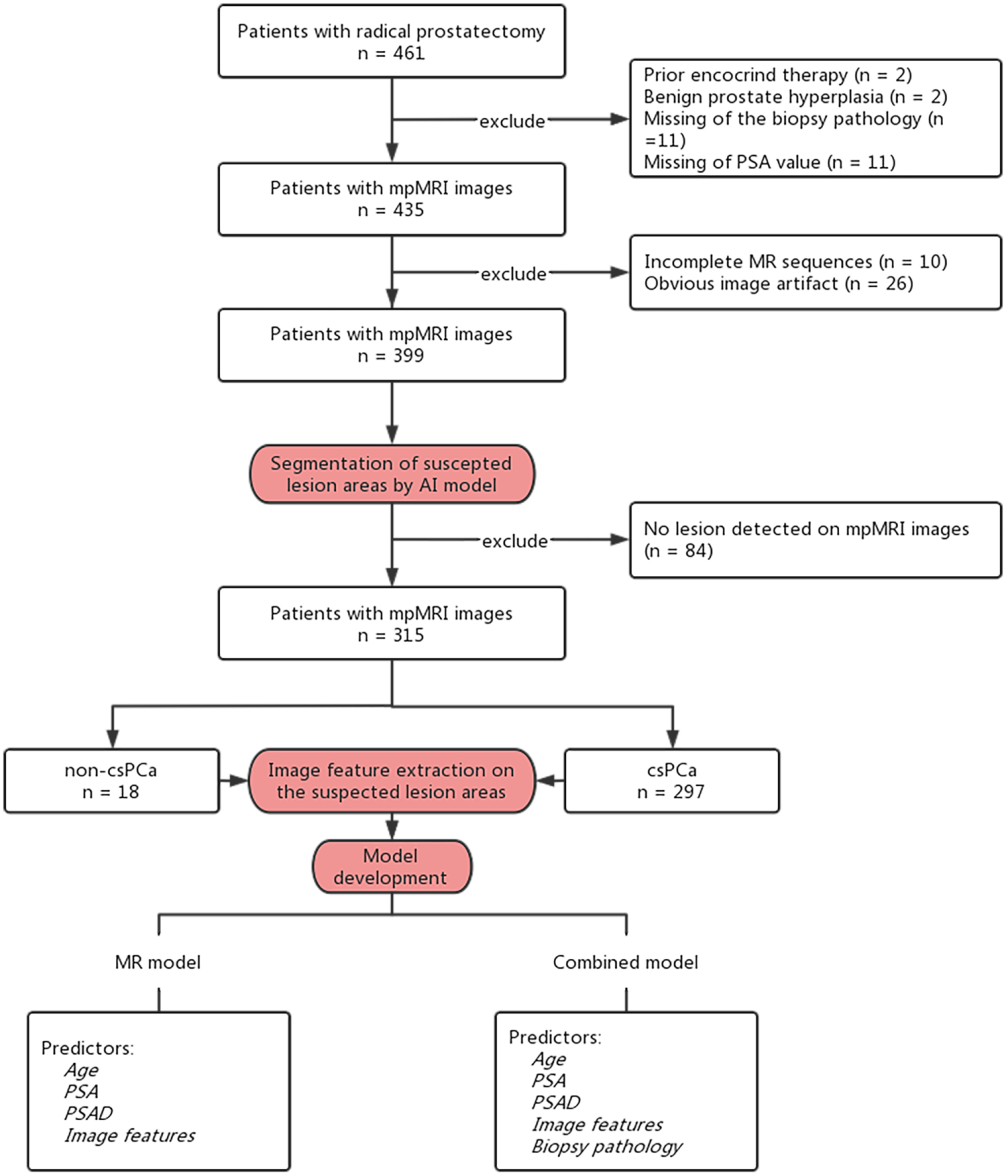
Data enrollment and research process. The study involved the collection of cases with comprehensive clinical and mpMRI images. A pre-trained AI model was employed to identify the regions of interest (ROI) corresponding to the suspected lesion areas. Subsequently, image features were extracted from the ROIs based on specific criteria. Finally, two prediction models, the MR model and the combined model, were trained using logistic regression

All patients underwent biopsy and RP with available pathology samples. The pathology was reviewed and reported by experienced pathologists according to the ISUP group. The reference standard was established based on the RP pathology results, with GS 3 + 3 = 6 considered non-csPCa and GS ≥ 3 + 4 considered csPCa. The data enrollment process is illustrated in Fig. 1.

### MR scanning protocols

The mpMRI images were obtained from three MR scanners, with 176 cases (55.9%) acquired from a 1.5 T scanner, 135 cases (42.9%) acquired from a 3.0T scanner, and 4 cases (1.2%) acquired from a 1.436T scanner. The transmit coils were body coils, and the receiver coils were phased array coils. No endorectal coil was used. Table 1 provides details on the MR scanners and imaging parameters.

**Table 1:** MR scanning protocols

Sequence	Parameters	MR scanner		
		Aera (1.5T, *n* = 176)	DISCOVERY MR750 (3.0T, *n* = 135)	uMR 586 (1.436T, *n* = 4)
DWI/ADC	b value (*10^6 s/mm^2^)	1000 [1000, 1000]	600 [500, 1000]	455 [405, 505]
	Repetition time (ms)	3000 [3000, 3230]	2080 [2080, 2100]	3520 [3520, 3520]
	Echo time (ms)	70.0 [70.0, 70.0]	59.7 [59.7, 59.8]	91.0 [91.0, 91.0]
	Echo train length	55.0 [55.0, 55.0]	1.00 [1.00, 1.00]	61.0 [61.0, 61.0]
	Pixel bandwidth (MHz)	1540 [1540, 1540]	1950 [1950, 1950]	1540 [1540, 1540]
	Reconstruction diameter (mm)	200 [200, 200]	340 [340, 340]	210 [210, 210]
	Slice thickness (mm)	3.50 [3.50, 3.50]	3.00 [3.00, 3.00]	4.00 [4.00, 4.00]
	Slice spacing (mm)	3.50 [3.50, 3.50]	4.00 [4.00, 4.00]	4.40 [4.40, 4.40]
	Pixel spacing (mm)	1.79 [1.79, 1.79]	1.33 [1.33, 1.33]	1.02 [1.02, 1.02]
T2WI	Repetition time (ms)	5540 [5540, 6000]	3470 [3370, 3760]	2200 [2200, 2200]
	Echo time (ms)	112 [112, 112]	105 [105, 107]	93.0 [93.0, 93.0]
	Echo train length	24.0 [24.0, 24.0]	16.0 [16.0, 16.0]	21.0 [21.0, 21.0]
	Pixel bandwidth (MHz)	200 [200, 200]	163 [163, 163]	180 [180, 180]
	Reconstruction diameter (mm)	200 [200, 200]	200 [200, 200]	200 [200, 200]
	Slice thickness (mm)	3.50 [3.50, 3.50]	3.00 [3.00, 3.00]	4.00 [4.00, 4.00]
	Slice spacing (mm)	3.50 [3.50, 3.50]	4.00 [4.00, 4.00]	4.40 [4.40, 4.40]
	Pixel spacing (mm)	0.625 [0.625, 0.625]	0.391 [0.391, 0.391]	0.439 [0.439, 0.439]

### Lesion segmentation by AI algorithms

The MR images were anonymized using self-developed software written in C +  + . The patient information in the DICOM file header was replaced with anonymous information using predefined rules. The software read the DICOM data, made the necessary modifications, and updated the original file to achieve complete anonymization.

After anonymization, the DICOM files were converted to the NIFTI format using the dicom2nii.py tool implemented in Python 3.5 and then input into our in-house deep learning-based AI model for the segmentation of suspicious PCa foci [4]. The functionalities of the AI model include automated selection of DWI and ADC images, segmentation of the prostate gland within the images, and further segmentation of suspicious prostate cancer regions. The segmented areas, identified as potentially cancerous by the AI model, were subsequently utilized for extracting image features in the next step. Notably, none of the cases in this study were previously used for training the AI model. Thus, in this study, the AI model was externally validated.

### Extraction of image features

**Fig. 2:**
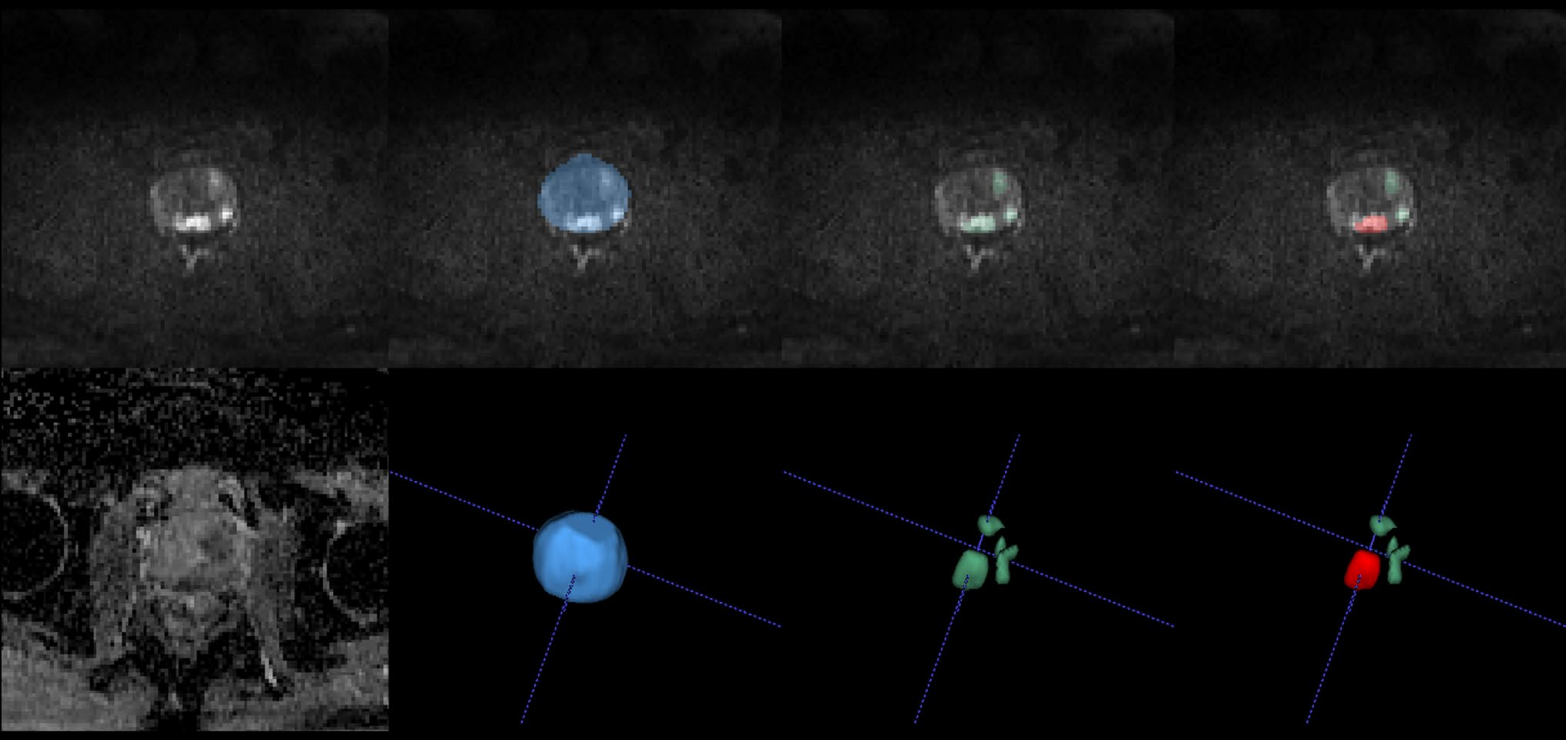
An example of AI segmented lesions and extraction of image features on mpMRI. A 74-year-old man with a serum PSA level of 7.93 ng/ml had mpMRI images showing multiple lesions on DWI (**a**) and ADC map (**b**). The DWI and ADC maps were automatically selected by the AI model, followed by segmentation of the prostate gland (blue area in (**c**) and (**d**)). Subsequently, the AI model segmented suspicious csPCa lesions on the DWI and ADC maps, as indicated by the green areas in (**e**) and (**f**). The largest lesion identified was designated the index lesion, represented by the red area in (**g**) and (**h**). Image features were then extracted specifically from the index lesion, which was classified as PI-RADS 4. Following a biopsy, pathology revealed non-csPCa with a Gleason score of 3 + 3. Subsequent pathology of the radical prostatectomy specimen showed csPCa, with a left lobe Gleason score of 4 + 4 = 8, accounting for approximately 10% of the gland, and a right lobe Gleason score of 4 + 3 = 7, accounting for approximately 7%

First, within the suspicious lesion regions segmented by the AI, the largest lesion is identified and defined as the index lesion. Subsequently, the following image features were calculated from the index lesion and used in the prediction model: (1) lesion volume, (2) mean apparent diffusion coefficient (ADC) value of the lesion (ADC_lesion_), (3) ADC value of the prostate outside the lesion (ADC_prostate_), (4) ratio of ADC value between the lesion and the prostate outside the lesion (ADC_lesion/prostate_), (5) mean signal intensity of the lesion on diffusion-weighted imaging (DWI) (DWI_lesion_), (6) DWI signal intensity of the prostate outside the lesion (DWI_prostate_), (7) signal intensity ratio of DWI between the lesion and the prostate outside the lesion (DWI_lesion/prostate_), (8) signal intensity ratio of the lesion between DWI and ADC (DWI_lesion_/ADC_lesion_), (9) the ratio of DWI_lesion/prostate_ to ADC_lesion/prostate_, and (10) the volume of the prostate gland. Moreover, the volume of the prostate gland was used to calculate the prostate-specific antigen density (PSAD), as shown in Fig. 2.

### Prediction model development

Two logistic regression models were established to forecast csPCa after RP: an MR model and a combined model. The MR model consisted of the predictor variables such as age, PSA, PSAD, and the ten types of MR image features. The combined model included biopsy pathology outcomes and the variables mentioned earlier. Univariate analysis was performed initially, followed by a forward and backward stepwise algorithm, which employed the Akaike information criterion (AIC) to select the variables for the final multivariable model.

### Model evaluation

The study evaluated the performance of three methods for predicting csPCa: biopsy pathology, MR model, and combined model. The evaluation was conducted using receiver operating characteristic (ROC) analysis, which calculates the area under the ROC curve (AUC). Decision curve analysis (DCA) compared each method’s clinical effects. Finally, a nomogram was created to visually display the performance of the prediction model.

### Statistical analysis

The statistical analysis was performed using R 4.1.3 software. Descriptive statistics were used to summarize the data, with mean (standard deviation) reported for continuous variables that followed a normal distribution and median [Q1, Q3] for continuous variables that did not follow a normal distribution. Categorical variables were reported as frequencies (percentage %).

The Shapiro‒Wilk test was employed to assess the normality of continuous variables. If the continuous variables followed a normal distribution, additional testing was conducted to examine the homogeneity of variances using an F test. If the variances were found to be homogeneous, the t test was utilized to compare features between the non-csPCa and csPCa groups, while one-way ANOVA was used to compare features among the ISUP groups of post-operation pathology. ADC_lesion_ was found to be applicable in this particular scenario. On the other hand, if the variances were not homogeneous, the corrected *t* test was applied to compare the features between the non-csPCa and csPCa groups, and the Kruskal‒Wallis test was used to compare the features among the ISUP groups of post-operation pathology. In this case, ADC_prostate_ and ADC_prostate/lesion_ were deemed applicable. For continuous variables that did not conform to a normal distribution, the Mann‒Whitney test was employed to compare the features between the non-csPCa and csPCa groups, and the Kruskal‒Wallis test was utilized to compare the features among the ISUP groups of post-operation pathology. The following variables in this study fell into this category: age, PSA, PSAD, prostate volume, lesion volume, DWI_lesion_, DWI_prostate_, DWI_lesion/prostate_, DWI_lesion_/ADC_lesion_, and DWI_lesion/prostate_/ADC_lesion/prostate_.

The Nagelkerke test was used to obtain the coefficient of determination (*R*^2^) values of the multivariable regression models. The DeLong test was used to compare the AUCs of the biopsy pathology, MR model, and combined model. A *P* value less than 0.05 was considered statistically significant.

## Results

### Clinical characteristics

A total of 315 patients were enrolled in this study. The average age of the patients was 70.8 ± 5.9. Among them, 42 (13.3%) patients underwent MRI examination after biopsy, with a median interval of 15 [3, 17] days. On the other hand, 273 (86.7%) patients underwent MRI examination before biopsy, with a median interval of 6 [4, 9] days. Of the 315 patients, 59 (18.7%) were diagnosed with non-csPCa by biopsy pathology, and 256 (81.3%) were diagnosed with csPCa. However, based on RP pathology, only 18 (5.7%) patients were diagnosed with non-csPCa, while 297 (94.3%) were diagnosed with csPCa.

Table 2 provides a summary of the patient characteristics stratified by biopsy pathology and RP pathology. The median PSA level was 8.5 [5.7, 14.2] ng/mL, and the median PSAD was 0.2 [0.1, 0.3] ng/mL/cm^3^. The median volume of the prostate gland was 35.6 [29.3, 42.3] cm^3^. In terms of biopsy pathology, the median number of biopsy cores was 12 [10, 14], and the median percentage of biopsy cores positive for cancer was 30% [10%, 60%]. Among the 256 patients diagnosed with csPCa by biopsy pathology, the majority had a Gleason score of 7 (*n* = 173, 67.6%), followed by Gleason score 6 (*n* = 75, 29.3%) and Gleason score 8–10 (*n* = 8, 3.1%).

**Table 2:** Clinical characteristics of the enrolled patients

	Overall	ISUP 1	ISUP 2	ISUP 3	ISUP 4	ISUP 5	*P* value
	(*N* = 315)	(*N* = 18)	(*N* = 90)	(*N* = 87)	(*N* = 46)	(*N* = 74)	
Age (years)							
Median [Q1, Q3]	71.0 [67.0, 75.0]	72.5 [66.3, 75.8]	69.5 [67.0, 75.0]	71.0 [67.0, 76.0]	71.5 [68.0, 75.8]	72.0 [67.0, 74.8]	0.621
PSA (ng/mL)							
Median [Q1, Q3]	12.1 [8.25, 20.4]	11.3 [9.78, 14.7]	10.7 [7.59, 17.0]	12.2 [7.83, 20.1]	11.1 [9.72, 17.6]	17.0 [11.1, 30.1]	< 0.001
PSAD (ng/mL/cm^3^)							
Median [Q1, Q3]	3.26 [2.05, 5.06]	2.47 [1.53, 3.40]	2.68 [1.83, 4.03]	3.05 [1.99, 4.73]	3.15 [2.10, 5.92]	4.66 [3.03, 7.19]	< 0.001
PI-RADS							
1	3 (1.0%)	0 (0%)	1 (1.1%)	1 (1.1%)	1 (2.2%)	0 (0%)	0.185
2	16 (5.1%)	3 (16.7%)	5 (5.6%)	5 (5.7%)	0 (0%)	3 (4.1%)	
3	120 (38.1%)	8 (44.4%)	41 (45.6%)	31 (35.6%)	12 (26.1%)	28 (37.8%)	
4	72 (22.9%)	0 (0%)	20 (22.2%)	20 (23.0%)	15 (32.6%)	17 (23.0%)	
5	104 (33.0%)	7 (38.9%)	23 (25.6%)	30 (34.5%)	18 (39.1%)	26 (35.1%)	
Biopsy pathology							
1	59 (18.7%)	13 (72.2%)	23 (25.6%)	20 (23.0%)	2 (4.3%)	1 (1.4%)	< 0.001
2	52 (16.5%)	2 (11.1%)	32 (35.6%)	14 (16.1%)	4 (8.7%)	0 (0%)	
3	71 (22.5%)	2 (11.1%)	25 (27.8%)	23 (26.4%)	10 (21.7%)	11 (14.9%)	
4	88 (27.9%)	1 (5.6%)	9 (10.0%)	26 (29.9%)	24 (52.2%)	28 (37.8%)	
5	45 (14.3%)	0 (0%)	1 (1.1%)	4 (4.6%)	6 (13.0%)	34 (45.9%)	
Lesion location							
PZ	59 (18.7%)	7 (38.9%)	18 (20.0%)	14 (16.1%)	11 (23.9%)	9 (12.2%)	0.131
TZ	76 (24.1%)	7 (38.9%)	24 (26.7%)	25 (28.7%)	6 (13.0%)	17 (23.0%)	
PZ + TZ	180 (57.1%)	4 (22.2%)	48 (53.3%)	48 (55.2%)	29 (63.0%)	48 (64.9%)	
Prostate volume (cm^3^)							
Median [Q1, Q3]	37.7 [29.5, 48.1]	43.7 [33.4, 66.3]	40.0 [31.3, 46.5]	41.1 [30.3, 52.3]	35.9 [29.1, 43.7]	34.4 [28.3, 44.8]	0.058
Lesion volume (cm^3^)							
Median [Q1, Q3]	2.05 [1.04, 4.48]	2.14 [0.998, 4.82]	1.82 [0.976, 4.12]	1.98 [1.02, 3.63]	2.02 [1.06, 4.76]	2.35 [1.21, 5.49]	0.606
ADC_lesion_							
Mean (SD)	0.857 (0.142)	0.903 (0.175)	0.879 (0.130)	0.856 (0.145)	0.860 (0.142)	0.818 (0.140)	0.093
ADC_prostate_							
Mean (SD)	1.34 (0.141)	1.31 (0.0884)	1.35 (0.136)	1.36 (0.135)	1.35 (0.150)	1.29 (0.147)	0.017
ADC_lesion/prostate_							
Mean (SD)	0.640 (0.0864)	0.685 (0.116)	0.652 (0.0843)	0.628 (0.0908)	0.634 (0.0714)	0.634 (0.0812)	0.123
DWI_lesion_							
Median [Q1, Q3]	0.779 [0.580, 2.54]	0.683 [0.492, 1.93]	0.719 [0.565, 2.58]	0.875 [0.623, 2.62]	1.44 [0.636, 2.52]	0.746 [0.480, 1.97]	0.102
DWI_prostate_							
Median [Q1, Q3]	0.489 [0.364, 1.45]	0.429 [0.279, 0.779]	0.474 [0.336, 1.57]	0.518 [0.410, 1.53]	0.784 [0.430, 1.35]	0.458 [0.262, 1.09]	0.112
DWI_lesion/prostate_							
Median [Q1, Q3]	1.65 [1.52, 1.86]	1.68 [1.53, 1.90]	1.60 [1.51, 1.80]	1.65 [1.51, 1.89]	1.71 [1.57, 1.93]	1.68 [1.50, 1.84]	0.194
DWI_lesion_/ADC_lesion_							
Median [Q1, Q3]	108 [69.5, 266]	70.2 [53.8, 230]	92.7 [68.4, 270]	138 [76.2, 280]	134 [84.9, 268]	104 [55.6, 208]	0.053
DWI_lesion/prostate_/ADC_lesion/prostate_							
Median [Q1, Q3]	2.60 [2.27, 3.06]	2.37 [2.18, 2.97]	2.55 [2.23, 2.89]	2.59 [2.30, 3.29]	2.75 [2.41, 3.18]	2.67 [2.19, 2.97]	0.134

*ADC_*lesion*_ mean ADC value of the lesion, *ADC*_*prostate*_ ADC value of the prostate outside the lesion, *ADC*_*lesion*/*prostate*_ ratio of ADC value between the lesion and the prostate outside the lesion, *DWI*_*lesion*_ mean signal intensity of the lesion on DWI, *DWI*_*prostate*_ DWI signal intensity of the prostate outside the lesion, *DWI*_*lesion*/*prostate*_ signal intensity ratio of DWI between the lesion and the prostate outside the lesion, *DWI*_*lesion*_/*ADC*_*lesion*_ signal intensity ratio of the lesion between DWI and ADC, *DWI*_*lesion*/*prostate*_/*ADC*_*lesion*/*prostate*_ the ratio of DWI_lesion/prostate_ to ADC_lesion/prostate_

Statistically significant differences were observed among the five RP ISUP groups (Table 2) in terms of PSA, PSAD, biopsy pathology, and ADC_prostate_ (all *P* < 0.05). However, no significant differences were observed in the other clinical and image features (all *P* > 0.05). In the comparison between the csPCa and non-csPCa groups (Table 3), significant differences were observed in terms of PSAD, PI-RADS score, biopsy pathology, and ADC_lesion/prostate_ (all *P* < 0.05). There were no significant differences observed in the other clinical and imaging features between the csPCa and non-csPCa groups (all *P* > 0.05).

**Table 3:** Odds ratios and their significance in the models

Feature	Comparison			Univariable model		MR model		Combined model	
	non-csPCa (*N* = 18)	csPCa (*N* = 297)	*P* value	OR	*P* value	OR	*P* value	OR	*P* value
Age (years)									
Median [Q1, Q3]	72.5 [66.3, 75.8]	71.0 [67.0, 75.0]	0.844	0.99 (0.91, 1.08)	0.863				
PSA (ng/mL)									
Median [Q1, Q3]	11.3 [9.78, 14.7]	12.2 [8.17, 20.8]	0.277	1.055 (1.001, 1.133)	0.096				
PSAD (ng/mL/cm^3^)									
Median [Q1, Q3]	2.47 [1.53, 3.40]	3.37 [2.09, 5.37]	0.025	1.373 (1.07, 1.903)	0.034	1.289 (1.024, 1.776)	0.077		
PI-RADS									
1	0 (0%)	3 (1.0%)	0.041	Reference					
2	3 (16.7%)	13 (4.4%)		0.00 (0.00-Inf)	0.998				
3	8 (44.4%)	112 (37.7%)		0.00 (0.00-Inf)	0.998				
4	0 (0%)	72 (24.2%)		1.00 (0.00-Inf)	> 0.999				
5	7 (38.9%)	97 (32.7%)		0.00 (0.00-Inf)	0.998				
Biopsy pathology									
1	13 (72.2%)	46 (15.5%)	< 0.001	Reference					
2	2 (11.1%)	50 (16.8%)		3.877 (0, inf)	0.994			2.647 (0, inf)	0.995
3	2 (11.1%)	69 (23.2%)		148.269 (0, inf)	0.992			254.63 (0, inf)	0.990
4	1 (5.6%)	87 (29.3%)		1322.67 (0, inf)	0.993			987.22 (0, inf)	0.993
5	0 (0%)	45 (15.2%)		157910.783 (0, inf)	0.991			202,620 (0, inf)	0.989
Lesion location									
PZ	7 (38.9%)	52 (17.5%)	0.071	Reference					
TZ	7 (38.9%)	173 (58.2%)		3.33 (1.12–9.92)	0.031				
PZ + TZ	4 (22.2%)	72 (24.2%)		2.42 (0.67–8.71)	0.175				
Prostate volume (cm^3^)									
Median [Q1, Q3]	43.7 [33.4, 66.3]	37.4 [29.3, 46.8]	0.112	0.993 (0.981, 1.009)	0.305				
Lesion volume (cm^3^)									
Median [Q1, Q3]	2.14 [0.998, 4.82]	2.04 [1.04, 4.43]	0.838	1.002 (0.966, 1.071)	0.922				
ADC_lesion_									
Mean (SD)	0.903 (0.175)	0.854 (0.140)	0.155	0.085 (0.003, 2.506)	0.159				
ADC_prostate_									
Mean (SD)	1.31 (0.0884)	1.34 (0.143)	0.371	3.912 (0.132, 117.516)	0.429				
ADC_lesion/prostate_									
Mean (SD)	0.685 (0.116)	0.638 (0.0838)	0.042	0.002 (0, 0.407)	0.025	0 (0, 0.001)	< 0.001	0 (0, 0.002)	0.001
DWI_lesion_									
Median [Q1, Q3]	0.683 [0.492, 1.93]	0.790 [0.589, 2.56]	0.160	1.391 (0.881, 2.409)	0.19			71.412 (1.884, 4162.15)	0.027
DWI_prostate_									
Median [Q1, Q3]	0.429 [0.279, 0.779]	0.494 [0.369, 1.47]	0.097	2.135 (0.913, 6.175)	0.112			0.003 (0, 1.331)	0.065
DWI_lesion/prostate_									
Median [Q1, Q3]	1.68 [1.53, 1.90]	1.65 [1.52, 1.86]	0.508	0.424 (0.153, 1.414)	0.111			0.003 (0, 0.076)	0.001
DWI_lesion_/ADC_lesion_									
Median [Q1, Q3]	70.2 [53.8, 230]	113 [71.1, 267]	0.060	1.003 (0.999, 1.009)	0.164	1.008 (1.002, 1.015)	0.017		
DWI_lesion/prostate_/ADC_lesion/prostate_									
Median [Q1, Q3]	2.37 [2.18, 2.97]	2.63 [2.28, 3.09]	0.316	0.838 (0.552, 1.479)	0.463	0.276 (0.123, 0.559)	0.001		

*ADC_*lesion*_ mean ADC value of the lesion, *ADC*_*prostate*_ ADC value of the prostate outside the lesion, *ADC*_*lesion*/*prostate*_ ratio of ADC value between the lesion and the prostate outside the lesion, *DWI*_*lesion*_ mean signal intensity of the lesion on DWI, *DWI*_*prostate*_ DWI signal intensity of the prostate outside the lesion, *DWI*_*lesion*/*prostate*_ signal intensity ratio of DWI between the lesion and the prostate outside the lesion, *DWI*_*lesion*_/*ADC*_*lesion*_ signal intensity ratio of the lesion between DWI and ADC, *DWI*_*lesion*/*prostate*_/*ADC*_*lesion*/*prostate*_ the ratio of DWI_lesion/prostate_ to ADC_lesion/prostate_

### Model development metrics

Table 3 summarizes the results of univariable and multivariable logistic regression analyses to identify the variables associated with csPCa. The predictor variables that were independently associated with csPCa and included in the MR model were PSAD, ADC_lesion/prostate_, DWI_lesion_/ADC_lesion_, and DWI_lesion/prostate_/ADC_lesion/prostate_. In the combined model, biopsy pathology, ADC_lesion/prostate_, DWI_lesion,_ DWI_prostate_, and DWIl_esion/prostate_ were included.

**Fig. 3:**
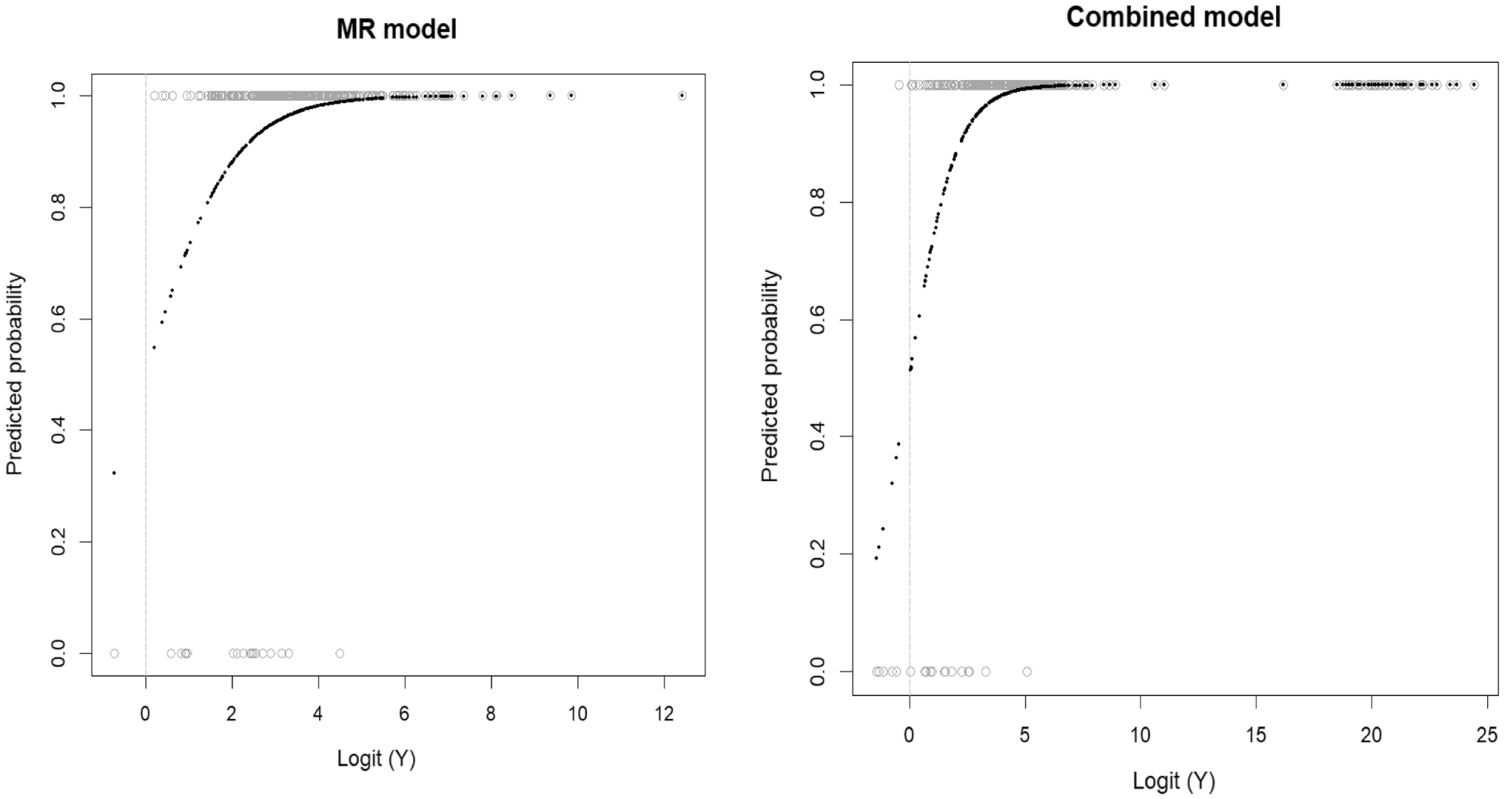
Visualization of the regression models. This plot represents the response curve of the logistic regression model, providing a visual representation of the results obtained from the generalized linear models of the MR model (**a**) and the combined model (**b**). The x-axis represents the logit transformation of the response variable (Y). The y-axis represents the predicted probability, showing the estimated probability of the response variable (Y) falling into the “success” category (such as csPCa or non-csPCa) based on the predictor variables used in the generalized linear model. This plot aids in visualizing the relationship between the predictor variables and the probability of the response, enabling a better understanding of the model’s behavior and its predictions. A Nagelkerke test was conducted, resulting in an MR model with an *R*^2^ of 0.219, indicating that it explains 21.9% of the variability in the outcome. On the other hand, the combined model had an *R*^2^ of 0.411, indicating that it explains 41.1% of the variability in the outcome

Figure 3 represents the response curve of the logistic regression model. The MR model had an *R*^2^ of 0.219, indicating that it explains 21.9% of the variability in the outcome. On the other hand, the combined model had an *R*^2^ of 0.411, indicating that it explains 41.1% of the variability in the outcome. These results suggest that both the MR model and the combined model can be useful in predicting csPCa. The combined model, which includes biopsy pathology and imaging features, had a higher *R*^2^ value and, thus, may provide more accurate predictions.

### Model evaluation

**Fig. 4:**
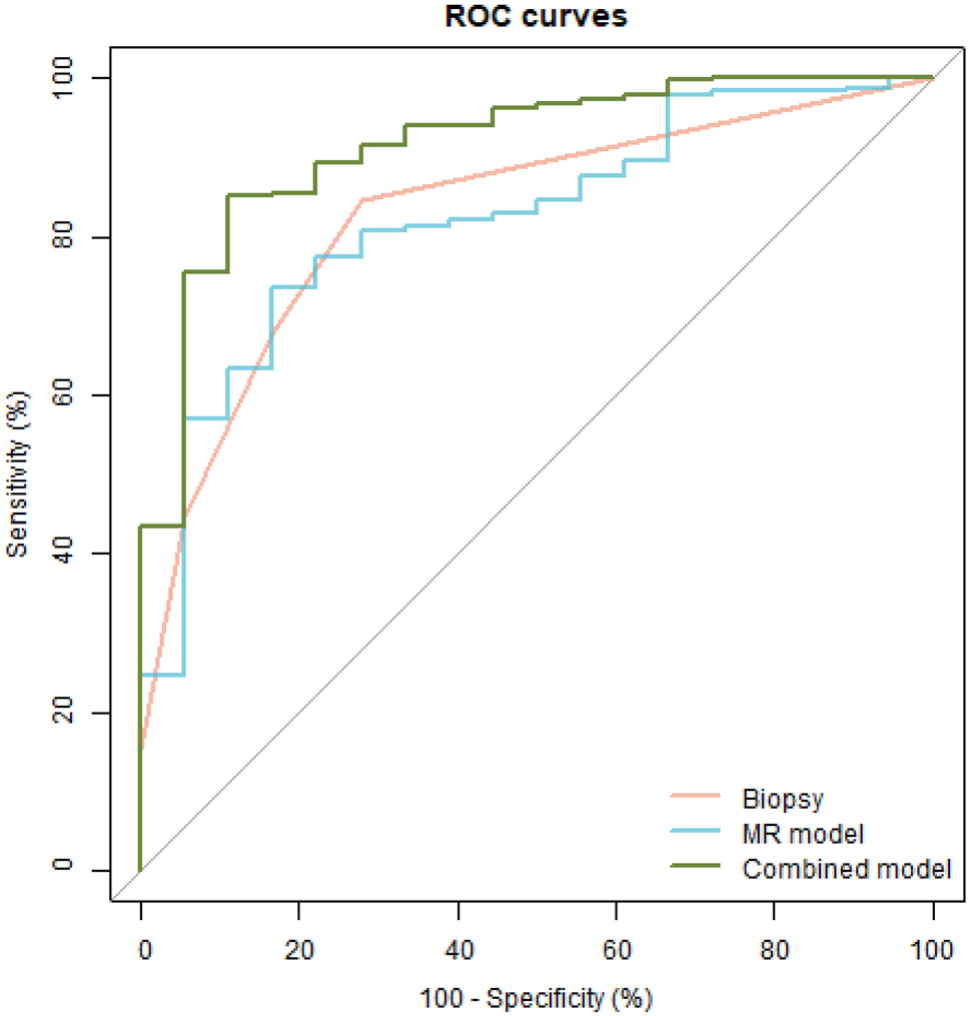
ROC curves of the models. This plot illustrates the ROC curves of various methods, with the red curve representing biopsy pathology, the blue curve representing the MR model, and the green curve representing the combined model. The AUC of the biopsy pathology was 0.820 (95% CI 0.728, 0.912), and the MR model had an AUC of 0.830 (95% CI 0.743, 0.916), with no significant difference observed between the two methods (*P* = 0.884). However, the AUC of the combined model (0.915, 95% CI 0.849, 0.980) was significantly higher than that of the biopsy ISUP (*P* = 0.042) and the MR model (*P* = 0.031)

The predictive performance of the biopsy pathology, MR model, and combined model were evaluated using ROC analysis, and the results are displayed in Fig. 4. To compare the classification ability of the same method for csPCa in different regions, we conducted separate analyses for the lesions located in the peripheral zone (PZ, *n* = 59), the transitional zone (TZ, *n* = 76), and lesions located across both the TZ and PZ (*n* = 180), in addition to the analysis of the entire cohort (*n* = 315). The evaluation metrics, including AUC, accuracy, sensitivity, specificity, positive predictive value, and negative predictive value, are presented in Table 4.

**Table 4:** Evaluation metrics of the different methods

Cohort	Evaluation metrics	Method		
		Biopsy pathology	MR model	Combined model
Overall (*N* = 315)	AUC	0.820 (0.728, 0.912)	0.830 (0.743, 0.916)	0.915 (0.849, 0.980)
	ACC	0.740 (0.738, 0.741)	0.838 (0.837, 0.839)	0.854 (0.853, 0.855)
	SEN	0.734 (0.684, 0.784)	0.845 (0.804, 0.886)	0.852 (0.811, 0.892)
	SPE	0.833 (0.661, 1.000)	0.722 (0.515, 0.929)	0.889 (0.744, 1.000)
	PPV	0.986 (0.971, 1.002)	0.980 (0.964, 0.997)	0.992 (0.981, 1.003)
	NPV	0.160 (0.086, 0.234)	0.220 (0.115, 0.326)	0.267 (0.155, 0.379)
Lesion in PZ (*N* = 59)	AUC	0.852 (0.718, 0.985)	0.863 (0.756, 0.969)	0.945 (0.881, 1.000)
	ACC	0.661 (0.654, 0.668)	0.780 (0.774, 0.785)	0.864 (0.861, 0.868)
	SEN	0.615 (0.483, 0.748)	0.769 (0.655, 0.884)	0.846 (0.748, 0.944)
	SPE	1.000 (1.000, 1.000)	0.857 (0.598, 1.000)	1.000 (1.000, 1.000)
	PPV	1.000 (1.000, 1.000)	0.976 (0.928, 1.023)	1.000 (1.000, 1.000)
	NPV	0.259 (0.094, 0.425)	0.333 (0.116, 0.551)	0.467 (0.214, 0.719)
Lesion in TZ (*N* = 76)	AUC	0.722 (0.415, 1.000)	0.889 (0.841, 0.937)	0.965 (0.915, 1.000)
	ACC	0.803 (0.799, 0.807)	0.789 (0.785, 0.794)	0.908 (0.906, 0.910)
	SEN	0.806 (0.714, 0.897)	0.778 (0.682, 0.874)	0.903 (0.834, 0.971)
	SPE	0.750 (0.326, 1.000)	1.000 (1.000, 1.000)	1.000 (1.000, 1.000)
	PPV	0.983 (0.950, 1.016)	1.000 (1.000, 1.000)	1.000 (1.000, 1.000)
	NPV	0.176 (− 0.005, 0.358)	0.200 (0.025, 0.375)	0.364 (0.079, 0.648)
Lesion in PZ and TZ (*N* = 180)	AUC	0.835 (0.733, 0.936)	0.772 (0.586, 0.958)	0.856 (0.710, 1.000)
	ACC	0.806 (0.804, 0.807)	0.861 (0.860, 0.862)	0.772 (0.770, 0.774)
	SEN	0.803 (0.744, 0.863)	0.873 (0.823, 0.922)	0.769 (0.706, 0.832)
	SPE	0.857 (0.598, 1.000)	0.571 (0.205, 0.938)	0.857 (0.598, 1.000)
	PPV	0.993 (0.979, 1.007)	0.981 (0.959, 1.002)	0.993 (0.978, 1.007)
	NPV	0.150 (0.039, 0.261)	0.154 (0.015, 0.293)	0.130 (0.033, 0.228)

*AUC area under the receiver characteristics curve, *ACC* accuracy, *SEN* sensitivity, *SPE* specificity, *PPV* positive predictive value, *NPV* negative predictive value

Within each method, we observed that there were no statistically significant differences in the AUC values when comparing the lesions located in the PZ (AUC_pz_), TZ (AUC_tz_), and PZ + TZ (AUC_pz+tz_) groups (all *P* < 0.05). The specific comparison of AUC values can be found in Table 5.

**Table 5:** Comparison of the AUCs in PZ, TZ, and PZ&TZ lesions in different methods

Method	AUC_pz_ vs. AUC_tz_	AUC_pz_ vs. AUC_pz+tz_	AUC_tz_ vs. AUC_pz+tz_
Biopsy pathology	0.661	0.409	0.236
MR model	0.451	0.845	0.497
Combined model	0.628	0.278	0.170

*AUC_*pz*_ AUC for evaluating csPCa lesions located in the peripheral zone, *AUC*_*tz*_ AUC for evaluating csPCa lesions located in the transitional zone, *AUC*_*pz+tz*_ AUC for evaluating csPCa lesions located across both the peripheral zone and the transitional zone

**Fig. 5:**
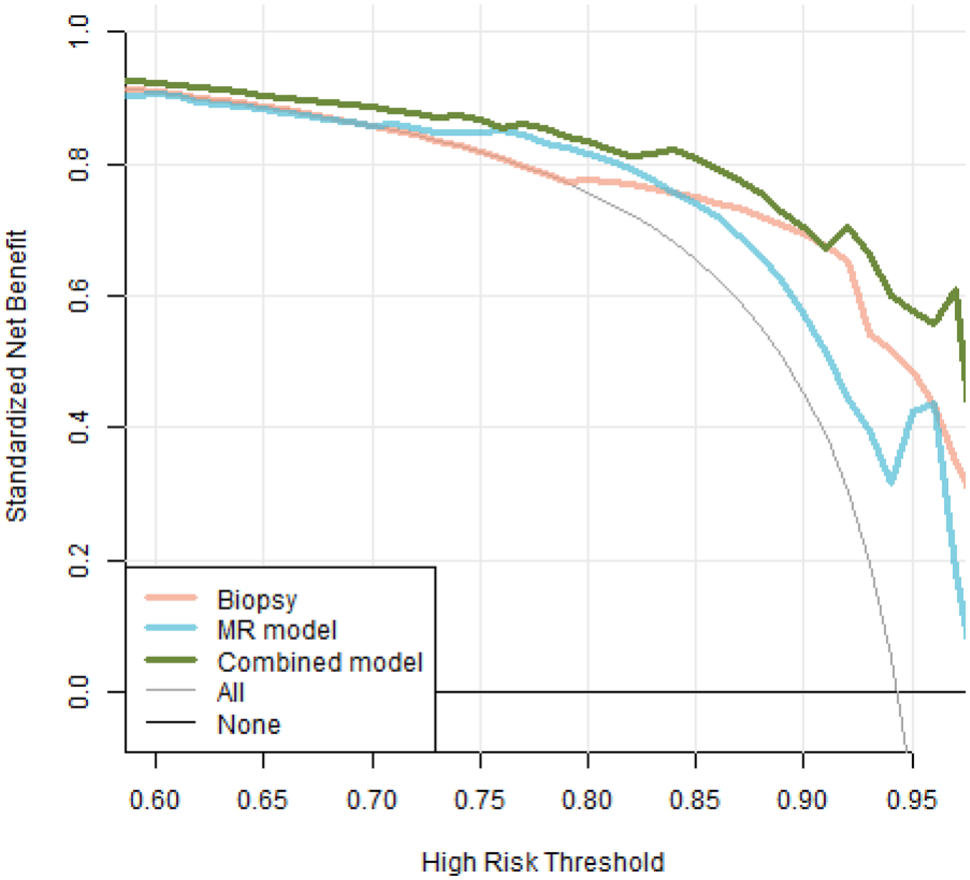
DCA curves of the models. This plot depicts the decision curve analysis of biopsy pathology (red), the MR model (blue), and the combined model (green), aiming to assess the clinical utility of these methods by analyzing the net benefit obtained from their use across various threshold probabilities. The “all” curve in the plot corresponds to the scenario where all patients are classified as positive (csPCa), irrespective of their actual diagnosis. Conversely, the “none” curve represents the scenario where no patients are classified as csPCa. The x-axis represents the threshold probability, which indicates the probability at which the methods are willing to act upon a positive prediction. The y-axis represents the net benefit gained from employing the models. By examining the decision curve plot, it can be concluded that the combined model outperformed both the biopsy pathology and MR model for all risk thresholds ranging from 0.5 to 1, indicating its superior clinical utility

**Fig. 6:**
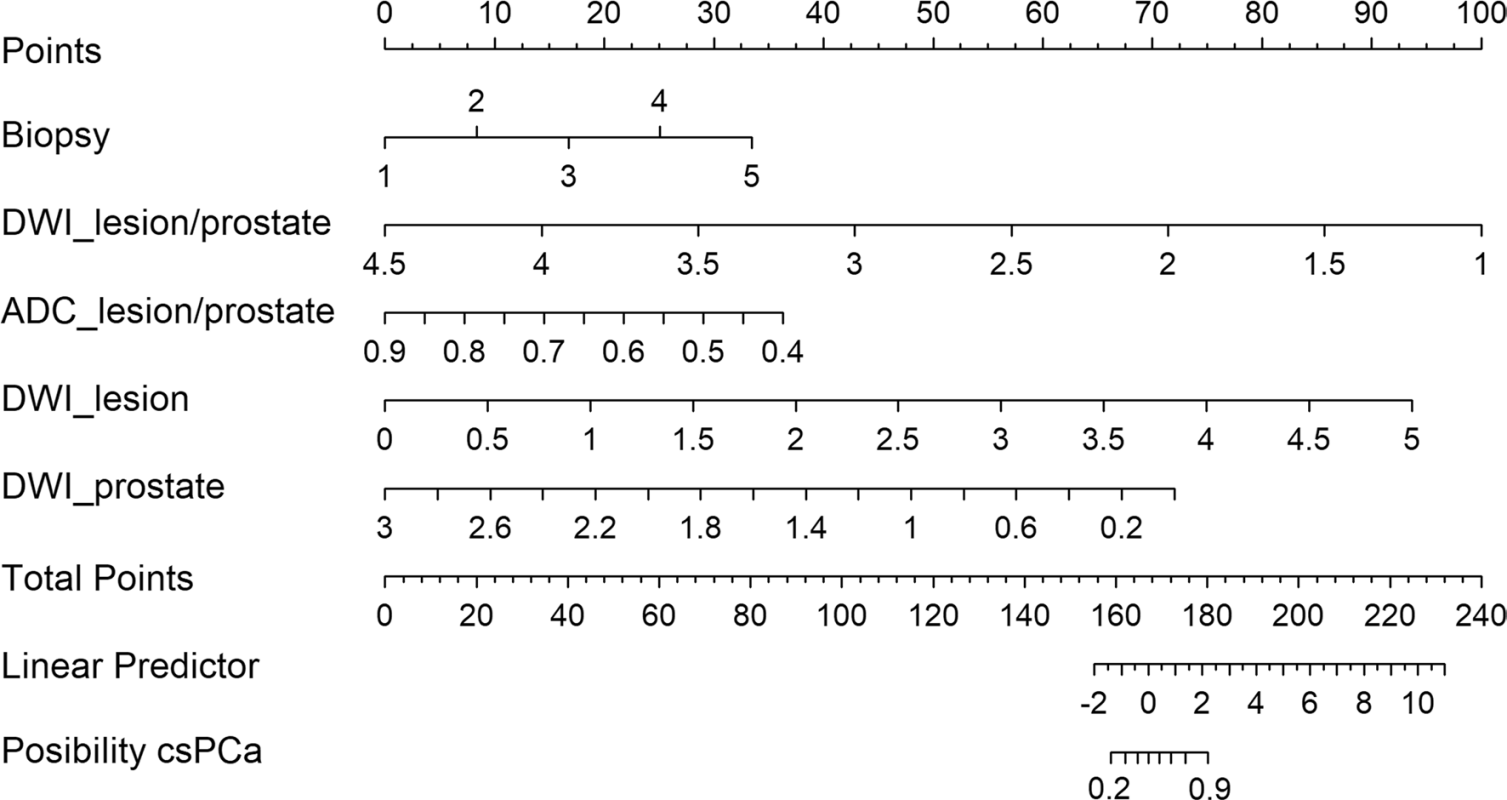
Nomogram of the combined model. This plot provides a visual representation of the combined model, which serves as a graphical tool for predicting the probability of csPCa based on the predictor variables. It allows for the estimation of an individual’s csPCa probability by assigning numerical values to each predictor variable and summing up the total points. This nomogram utilizes the uppermost line as a reference for scoring points ranging from 0 to 100, corresponding to each predictor. Predictor variables, including biopsy pathology and AI-extracted image features (DWI_lesion/prostate_, ADC_lesion/prostate_, DWI_lesion_, DWI_prostate_), are displayed below with bars indicating their relative weight. The sum of points can be checked on the “Points” line, and the corresponding probability of csPCa can be ascertained from the lowermost line

In terms of overall performance, the AUC of the biopsy pathology was 0.820 (95% CI 0.728, 0.912), and the MR model had an AUC of 0.830 (95% CI 0.743, 0.916), with no significant difference observed between the two methods (*P* = 0.884). However, the AUC of the combined model (0.915, 95% CI 0.849, 0.980) was significantly higher than that of the biopsy ISUP (*P* = 0.042) and the MR model (*P* = 0.031). The results of DCA, presented in Fig. 5, indicated that the combined model was superior to the biopsy pathology and MR model for all risk thresholds from 0.5 to 1. To further illustrate the predictive efficacy of the best model, a nomogram was created and is shown in Fig. 6.

## Discussion

The risk of PCa is stratified based on the ISUP grade group from pathology. Preoperative pathology is typically obtained through biopsy. However, discrepancies between biopsy pathology and post-operative pathology can result in under- or overestimation of prostate cancer risk levels [11]. In this study, we proposed that mpMRI can aid in the identification of csPCa in patients initially diagnosed with non-csPCa by biopsy. An MR model and a combined model were developed using mpMRI image features to predict the presence of csPCa in post-operation pathology. The efficacy of both models was compared to biopsy pathology alone. The results demonstrated that the combined model had a significantly higher AUC than both biopsy pathology and the MR model.

Currently, a biopsy is considered the gold standard for diagnosing prostate cancer. The EAU guidelines on prostate cancer recommend combining targeted biopsy (TB) with systematic biopsy (SB) as the first-line biopsy method in patients diagnosed with PCa with an abnormal MRI [12]. Prostate MRI utilizes the PI-RADS scoring system to categorize patients who are candidates for biopsy on a 1-to-5 risk scale for csPCa. The main objective of PI-RADS is to establish a standardized and consistent approach for evaluating prostate mpMRI scans in the detection of csPCa. As research on PI-RADS has advanced, it has become evident that higher PI-RADS scores correspond to an increased likelihood of csPCa. Nonetheless, there is a lack of sufficient research dedicated to reassessing PI-RADS scores in conjunction with pathological findings after biopsy. Further investigation is required in this area to ascertain the effectiveness of PI-RADS scoring in assessing csPCa in patients after biopsy.

Prostate MRI and related MRI-directed biopsies have been shown to be at least as diagnostically effective as systematic biopsies alone in diagnosing significant cancers. Studies have proven that the concordance between biopsy and prostatectomy grading was highest in combined biopsy (CB) but still with misdiagnosis of csPCa in 25% of men [13]. Thus, we suggest that mpMRI should be re-evaluated after biopsy to compensate for the limitations of biopsy pathology. In this study, we propose two models that might have potential for three applications in the future. The first application is in the initial biopsy for prostate cancer, where the MR model can determine whether another biopsy is necessary if the biopsy results are negative. If the MR model predicts a low likelihood of csPCa, observation may be an option, but if the MR model predicts a high likelihood of csPCa, another biopsy is recommended. The second application is in cases where the biopsy result is non-csPCa, where the combined model results can be used as a reference. If the combined model predicts a low likelihood of csPCa, conservative treatment may be appropriate, but if it predicts a high likelihood, more aggressive treatment is recommended. The third application is in active surveillance of PCa patients, where measures can be taken to monitor patients according to the results of the MR model or the combined model.

In the PI-RADS system, DWI and ADC image features are utilized for the detection of csPCa, including the typical observations of significantly high DWI signal and significantly low ADC values. In this study, we transformed these descriptive features into computed image feature values. For example, a higher DWI signal intensity (DWI_lesion_) and a higher contrast ratio between the DWI signal intensity of the lesion and the background signal intensity (DWI_lesion/prostate_) indicate a more prominent display of the lesion on the DWI image. Similarly, a lower ADC value (ADC_lesion_) and a lower contrast ratio between the ADC value of the lesion and the background ADC value (ADC_lesion/prostate_) result in a more distinct display of the lesion on the ADC map. These types of image features have been widely used in previous studies and have demonstrated their value in indicating tumor invasiveness. Previous studies have found that there is a negative correlation between DWI signal intensities, ADC values and Gleason score, indicating that higher DWI signal intensities and lower ADC values are associated with higher Gleason scores and more aggressive prostate cancer [14–16]. The researchers suggest that ADC values can be used as a non-invasive biomarker to aid in the diagnosis and management of prostate cancer.

However, manually calculated ADC values can vary due to several factors, such as differences in the region of interest (ROI) placement, differences in the b-values used for calculation, and differences in the software used for calculation [17]. Furthermore, there is no agreed ADC tumor cutoff value that could be reliably used to determine abnormally low ADC within a lesion [14, 15]. Therefore, the potential of DWI and ADC for evaluating the aggressiveness of PCa is limited to theoretical use and not practical application in clinical settings. Our study confirms the relationship between features in ADC and DWI images and the aggressiveness of PCa, which is similar to previous research. However, we have three additional advantages. First, we employed an AI model to automatically segment the suspected areas of PCa, which eliminates human intervention. This reduces the burden on doctors and guarantees the consistency of feature extraction [18]. Second, unlike in previous studies where AI models were mainly used for pre-biopsy diagnosis, the AI model in this study was utilized for post-biopsy re-evaluation. Third, we developed an objective prediction model based on a nomogram that outputs the probability of csPCa, which provides doctors with an intuitive reference. This model has the potential to be a valuable tool for urologists’ decision making once it has been fully validated [19].

Our study has some limitations. The first limitation of this study is that the data were collected from a single institution and were not obtained prospectively. This limits the generalizability of the study findings to other settings and populations, and the retrospective nature of the data collection can introduce bias and confounding factors. Thus, caution should be exercised when interpreting the results of this study, and further studies are needed to validate the findings in larger and more diverse populations. The second limitation of this study is that only patients who underwent radical prostatectomy were enrolled, as RP pathology was required for the analysis. However, in the broader clinical context, many patients may not be candidates for RP due to various reasons, such as advanced prostate cancer with no chance for curative surgery. Therefore, the generalizability of the study’s conclusions should be further assessed in patient populations that do not undergo RP. Future studies could include patients who undergo alternative treatments or active surveillance to assess the performance of the AI model in these populations. The study has a limitation in that only a limited number of clinical variables were included, which were age, PSA, and mpMRI. Other important clinical and imaging data, such as digital rectal examination, ultrasound, PET-CT, and prostate health index (PHI), were not taken into account. Therefore, incorporating a broader range of relevant data in future studies is necessary to enhance the precision and dependability of the prediction model.

In summary, AI-extracted image features from mpMRI images can accurately predict the aggressiveness of prostate cancer, similar to biopsy pathology. The accuracy of this prediction can be further improved by combining the AI-extracted mpMRI image features with biopsy pathology, which outperforms biopsy pathology alone. After further evaluation, this prediction model can be used for the re-evaluation of biopsy pathology and active surveillance of prostate cancer.
